# Study design for development of novel safety biomarkers of drug-induced liver injury by the translational safety biomarker pipeline (TransBioLine) consortium: a study protocol for a nested case–control study

**DOI:** 10.1186/s41512-023-00155-z

**Published:** 2023-09-12

**Authors:** Jane I. Grove, Camilla Stephens, M. Isabel Lucena, Raúl J. Andrade, Sabine Weber, Alexander Gerbes, Einar S. Bjornsson, Guido Stirnimann, Ann K. Daly, Matthias Hackl, Kseniya Khamina-Kotisch, Jose J. G. Marin, Maria J. Monte, Sara A. Paciga, Melanie Lingaya, Shiva S. Forootan, Christopher E. P. Goldring, Oliver Poetz, Rudolf Lombaard, Alexandra Stege, Helgi K. Bjorrnsson, Mercedes Robles-Diaz, Dingzhou Li, Thi Dong Binh Tran, Shashi K. Ramaiah, Sophia L. Samodelov, Gerd A. Kullak-Ublick, Guruprasad P. Aithal, on behalf of the TransBioLine consortium

**Affiliations:** 1 Nottingham Digestive Diseases Centre, Translational Medical Sciences, School of Medicine, University of Nottingham, Nottingham, NG7 2UH UK; 2 NIHR Nottingham Biomedical Research Centre, Nottingham University Hospitals NHS Trust and the University of Nottingham, Nottingham, UK; 3 Servicios de Aparato Digestivo Y Farmacologia Clínica, Instituto de Investigación Biomédica de Málaga-IBIMA Plataforma Bionand, Hospital Universitario Virgen de La Victoria, Universidad de Málaga, Malaga, Spain; 4 Centro de Investigación Biomédica en Red de Enfermedades Hepáticas Y Digestivas (CIBERehd), Madrid, Spain; 5 Department of Medicine II, University Hospital, LMU Munich, Munich, Germany; 6 Department of Gastroenterology, Landspitali University Hospital Reykjavik, University of Iceland, Reykjavík, Iceland; 7 Faculty of Medicine, University of Iceland, Reykjavík, Iceland; 8 University Clinic for Visceral Surgery and Medicine, University Hospital Inselspital and University of Bern, Bern, Switzerland; 9 Translational and Clinical Research Institute, Newcastle University, Newcastle Upon Tyne, NE2 4HH UK; 10 TAmiRNA GmbH, Vienna, Austria; 11 Experimental Hepatology and Drug Targeting (HEVEPHARM), Institute of Biomedical Research of Salamanca (IBSAL), University of Salamanca, Salamanca, Spain; 12 Worldwide Research Development and Medical, Pfizer, NY USA; 13 Centre for Drug Safety Science, University of Liverpool, Liverpool, UK; 14 SIGNATOPE GmbH, 72770 Reutlingen, Germany; 15 ABX-CRO Advanced Pharmaceutical Services, Forschungsgesellschaft mbH, Cape Town, 7441 South Africa; 16 Charité–Universitätsmedizin Berlin, Central Biobank Charité (ZeBanC), Berlin, Germany; 17 Division of Gastroenterology and Hepatology, Department of Internal Medicine, Sahlgrenska University Hospital, Gothenburg, Sweden; 18 Department of Clinical Pharmacology and Toxicology, University Hospital Zurich, University of Zurich, 8006 Zurich, Switzerland; 19 Mechanistic Safety, CMO & Patient Safety, Global Drug Development, Novartis Pharma, 4056 Basel, Switzerland

## Abstract

A lack of biomarkers that detect drug-induced liver injury (DILI) accurately continues to hinder early- and late-stage drug development and remains a challenge in clinical practice. The Innovative Medicines Initiative’s TransBioLine consortium comprising academic and industry partners is developing a prospective repository of deeply phenotyped cases and controls with biological samples during liver injury progression to facilitate biomarker discovery, evaluation, validation and qualification.

In a nested case–control design, patients who meet one of these criteria, alanine transaminase (ALT) ≥ 5 × the upper limit of normal (ULN), alkaline phosphatase ≥ 2 × ULN or ALT ≥ 3 ULN with total bilirubin > 2 × ULN, are enrolled. After completed clinical investigations, Roussel Uclaf Causality Assessment and expert panel review are used to adjudicate episodes as DILI or alternative liver diseases (acute non-DILI controls). Two blood samples are taken: at recruitment and follow-up. Sample size is as follows: 300 cases of DILI and 130 acute non-DILI controls. Additional cross-sectional cohorts (1 visit) are as follows: Healthy volunteers (*n* = 120), controls with chronic alcohol-related or non-alcoholic fatty liver disease (*n* = 100 each) and patients with psoriasis or rheumatoid arthritis (*n* = 100, 50 treated with methotrexate) are enrolled. Candidate biomarkers prioritised for evaluation include osteopontin, glutamate dehydrogenase, cytokeratin-18 (full length and caspase cleaved), macrophage-colony-stimulating factor 1 receptor and high mobility group protein B1 as well as bile acids, sphingolipids and microRNAs. The TransBioLine project is enabling biomarker discovery and validation that could improve detection, diagnostic accuracy and prognostication of DILI in premarketing clinical trials and for clinical healthcare application.

## Introduction

Idiosyncratic drug-induced liver injury (DILI) is an unpredictable and serious adverse event with an annual incidence estimated to be 2.7, 19.1 and 23.8 per 100,000 in the USA, Iceland and China, respectively [1–3]. However, in people taking many common medications, the incidence is substantially higher, for example in 156 per 100,000 users of amoxicillin-clavulanate and 5.5% among individuals treated with an antituberculosis fixed-dose combination therapies [4, 5]. Although rare, DILI accounted for 11% of acute liver failure (ALF) cases in the USA from 1998 to 2013 [6]. DILI occurs in association with a large number of drugs, and emerging immunotherapy regimens devised for cancer treatment are increasingly reported to be associated with increased risk of DILI development [7]. Furthermore, DILI shows heterogeneity in presentation with different phenotypic categories described, which can be indistinguishable from other causes of liver injury [8].

Crucially, DILI has substantial impact during drug development resulting in compound attrition, project termination, prescribing restrictions and/or withdrawal of promising innovative medicines [9–12]. A major hurdle is a lack of regulatory-qualified safety biomarkers to enable robust prediction of clinical safety events from preclinical data, assessment of risk in individual patients and prognosis of clinical outcome, in addition to a limited mechanistic understanding of toxicity. The low incidence of DILI means that it may only be identified when a large number of people have been exposed to the drug. Furthermore, the lack of specific tests can result in a delayed or missed diagnosis [13, 14].

Recently, the Innovative Medicines Initiative’s (IMI) Safer and Faster Evidence-based Translation (SAFE-T) consortium and others have identified novel safety candidate biomarkers for DILI [15], which have received regulatory support from the US Food and Drug Administration (FDA) [16]. However, none of the new candidate biomarkers has achieved full qualification yet; further confirmatory data are needed to bolster the initial biomarker datasets to complete the qualification process, necessitating additional research studies [17]. This will permit context-of-use evaluation of diagnostic (distinction of DILI from alternative diagnoses such as autoimmune, viral and ischaemic hepatitis) and prognostic biomarkers (predictors of development of jaundice, ALF, transplantation and death).

Regulatory acceptance of safety biomarkers for a defined use in drug development requires large-scale collaboration since DILI is rare and substantial sample sizes are needed to ensure case and control numbers are sufficient to test new biomarkers for qualification. In order to develop a deeply characterised and comprehensively analysed cohort sufficient for robust analysis, the TransBioLine consortium, a network of leading European research and pharmaceutical institutions and small-medium enterprises, has been established with a dedicated work package to deliver this task, outlined in this protocol.

We describe here the study protocol design to evaluate, validate and qualify candidate DILI safety biomarkers suitable for use in premarketing clinical trials and for clinical application.

## Methods and analysis

### Objectives

The primary aim is to evaluate diagnostic accuracy of candidate blood biomarkers in identifying DILI cases. The secondary aim is to evaluate the accuracy of candidate blood biomarkers in prognostication of DILI. This will entail assessment and characterization of patients with acute liver injury and grouping them into those specifically attributed to DILI, distinct from those due to alternate aetiology (‘acute non-DILI’). Other control groups enrolled are healthy volunteers (HV), patients with chronic liver disease (alcohol-related and non-alcoholic fatty liver diseases; ARLD and NAFLD) and chronic systemic diseases (psoriasis and rheumatoid arthritis). We will collect biological samples and follow-up the acute liver injury patients to enable subsequent clinical categorisation of the disease aetiology and progression/outcomes (recovery, death or transplantation). Biomarkers on their own, or in combination, which are able to distinguish DILI from competing diagnoses, as well as those able to identify DILI cases with adverse prognosis (i.e. those who progress to death or transplantation), will be validated. Finally, biomarker panels will be put forward for qualification by regulatory authorities in the particular context of use.

As a secondary objective, this study will generate a repository of comprehensive biomarker data, patient phenotype information and biological samples that can facilitate future qualifications of other or refined contexts of use or additional biomarker candidates.

### Study design

**Fig. 1:**
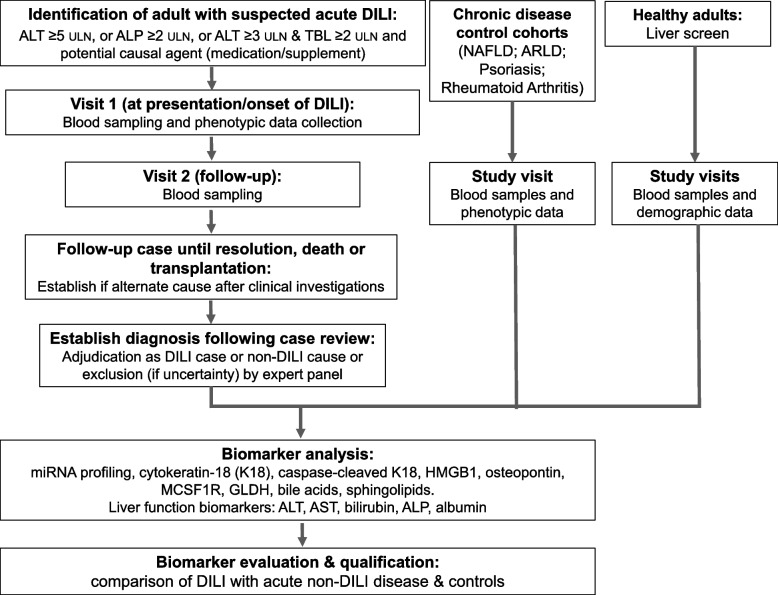
Study overview. Participants will be recruited to cohorts based on criteria detailed in Table 1. ARLD, alcohol-related fatty liver disease; ALP, alkaline phosphatase; ALT, alanine transaminase; K18, cytokeratin-18; DILI, drug-induced liver injury; GLDH, glutamate dehydrogenase; HMGB1, high mobility group box 1; MCSF1R, macrophage colony-stimulating factor 1 receptor; NAFLD, non-alcoholic fatty liver disease; TBL, total bilirubin; ULN, upper limit of normal

A nested case–control observational study design has been devised to prospectively identify and enroll a cohort of patients with acute liver injury at presentation at secondary care centres in six European countries through attendance for standard clinical care pathways (Fig. 1 and Table 1). Patients are assessed clinically and through investigations (laboratory tests and imaging) and sub-grouped as DILI (‘cases’) or acute ‘non-DILI’ (‘controls’). Both cases and controls are followed up to recovery or death or transplantation. Blood samples are obtained from both groups on two occasions (at presentation and on follow-up). In addition, we enroll HV, patients with ARLD or NAFLD or systemic diseases (psoriasis and rheumatoid arthritis) as further control groups and obtain samples from them (1 visit). These control cohorts are included to establish utility of candidate biomarkers in distinguishing DILI from alternate common liver injuries and to establish reference intervals.

**Table 1:** Recruiting centres opened and ethical approvals

Centre	Location	Ethical approval
Addenbrooke’s University Hospital, Cambridge University Hospitals NHS Trust Hospital	Cambridge, UK	UK Health Research Authority (Refs.: 15/YH/0294; GM010201; 14/EM/0145)
Hampshire Hospitals NHS Foundation Trust	Basingstoke, UK	
Newcastle upon Tyne Hospitals NHS Foundation Trust	Newcastle upon Tyne, UK	
Ninewells Hospital, Tayside NHS Trust	Dundee, UK	
Nottingham University Hospitals NHS Trust	Nottingham, UK	
Queen Alexandra Hospital, Portsmouth Hospitals NHS Trust	Portsmouth, UK	
Royal Cornwall Hospitals NHS Trust	Treliske, UK	
Royal Free Hospital	London, UK	
Royal Sussex County Hospital, Brighton and Sussex University Hospitals NHS Trust	Brighton, UK	
University Hospitals Birmingham	Birmingham, UK	
University Hospitals Trust Southampton	Southampton, UK	
Landspitali University Hospital	Reykjavik, Iceland	Bioethics Committee Iceland (Ref.: 15–104-V1)
Malaga University Hospital	Malaga, Spain	Biomedical Investigation Ethics Committee of Andalucia (Ref.: AND-HEP-2015–01)
Munich University Hospital	Munich, Germany	Ethical Commission of Ludwig Maximilian University of Munich (Project 85–16)
Sahlgrenska University Hospital	Gothenburg, Sweden	Swedish Ethics Review Authority (Ref.: 2022–04078-01)
University Hospital Bern	Bern, Switzerland	Bern Cantonal Ethics Committee for Research (Ref.: 2016–00932)
University Hospital Zurich	Zurich, Switzerland	Zurich Cantonal Ethics Committee (Ref.: 2019–01451)
Pfizer Global Research & Development	Brussels, Belgium	Ethics Committee Erasme Hospital (Ref.: P2019/530)

### Participants and recruitment

Ethical approvals were obtained from local ethical review authorities for each of the recruiting locations (Table 1). The consortium also includes an ethical advisory board to oversee policies and activities. Studies are conducted according to the Declaration of Helsinki (Hong Kong Amendment) and Good Clinical Practice (European guidelines) with all participants providing written informed consent or with written informed consent from a personal consultee in specified circumstances when participants lack capacity to give informed consent. The inclusion and exclusion criteria for each study cohort are detailed in Table 2. The recruitment period is planned from October 2019 to April 2024. The sample size was determined in consultation with the funder, IMI.

**Table 2:** Recruitment eligibility criteria for each cohort

**Cohorts**	**DILI** *n* = *30* *(2 visits)*	**Other acute liver injury (non-DILI)** *n* = *13 (2 visits)*	**Chronic liver disease (ARLD/NAFLD)** *n* = *20 (1 visit)*	**Rheumatoid arthritis and/or psoriasis** *n* = *100 (1 visit)*	**Healthy volunteers (HV)** *n* = *120* *(1 visit)*
**Inclusion criteria**	Age ≥ 18	Age ≥ 18	Age ≥ 18	Age ≥ 18	Age ≥ 18
	Exposure to potential causal agent	Exposure to potential causal agent	Clinical diagnosis of ARLD/NAFLD	Diagnosed with rheumatoid arthritis or psoriasis for ≥ 2 years	BMI ≤ 32
	Meets one of the following analytical thresholds at enrolment (visit 1)	Meets one of the following analytical thresholds at enrolment (visit 1)	ARLD: current > 112 g alcohol per week or past sustained alcohol excess clinically suggested as contributing to disease	Eligible for methotrexate treatment	Normal liver enzymes
	○ ALT ≥ 5 × ULN	○ ALT ≥ 5 × ULN			
	○ ALP ≥ 2 × ULN	○ ALP ≥ 2 × ULN			
	○ ALT ≥ 3 × ULN + TBL > 2 × ULN	○ ALT ≥ 3 × ULN + TBL > 2 × ULN			
	Diagnosis of acute DILI by panel	Diagnosis of non-DILI acute liver injury by panel	NAFLD:≤ 112 g alcohol per week	Either treated with methotrexate for past 6 months or unexposed to methotrexate in past 24 months	
**Exclusion criteria**	Clear diagnosis of alternative acute liver condition (e.g. acute viral or autoimmune hepatitis unrelated to the drug)	Relapse of previously diagnosed chronic liver injury	Diagnosis of other liver disease	Diagnosis of liver disease other than ARLD/NAFLD	Diagnosis of liver disease
			Suspicion of acute cause of liver injury (e.g. viral hepatitis, autoimmune hepatitis, DILI)	Other significant dermatological or rheumatological condition	Transient Elastography CAP score of ≥ 260 dB/m

*Abbreviations*: *ARLD* Alcohol-related liver disease, *ALP* Alkaline phosphatase, *ALT* Alanine transaminase, *CAP* Controlled attenuation parameter, *DILI* Drug-induced liver injury, *HV* Healthy volunteers, *NAFLD* Non-alcoholic fatty liver disease, *TBL* Total bilirubin, *ULN* Upper limit of normal. Italics denotes planned group size. Headings are in bold

Since DILI is rare and there are no established biomarkers, the cohort size was derived based on feasibility of enrolling cases of DILI as well as acute non-DILI controls within the study period and was informed by previous studies [15]. Based on previous biomarker data comparing DILI with HV [15], our cohort would be powered to detect a 20% change in caspase-cleaved cytokeratin-18 (ccK18), a 10% change in macrophage colony-stimulating factor 1 receptor (MCSF1R) and an 18% change in osteopontin (OPN). In addition, we have carried out power analysis using area under the receiver operator characteristic (ROC) curves (based on previous studies [15]) with assumption that 10% of cases will have outcome of death/transplantation. Using a sample size of 100 DILI cases and 50 healthy volunteers, which would be appropriate for a learning phase analysis (which could then be followed by a validation phase), the minimum power across all of the candidate biomarkers for diagnosis or prognosis is 0.88.

### Suspected DILI cases (subsequently sub-divided into DILI and non-DILI)

**Fig. 2:**
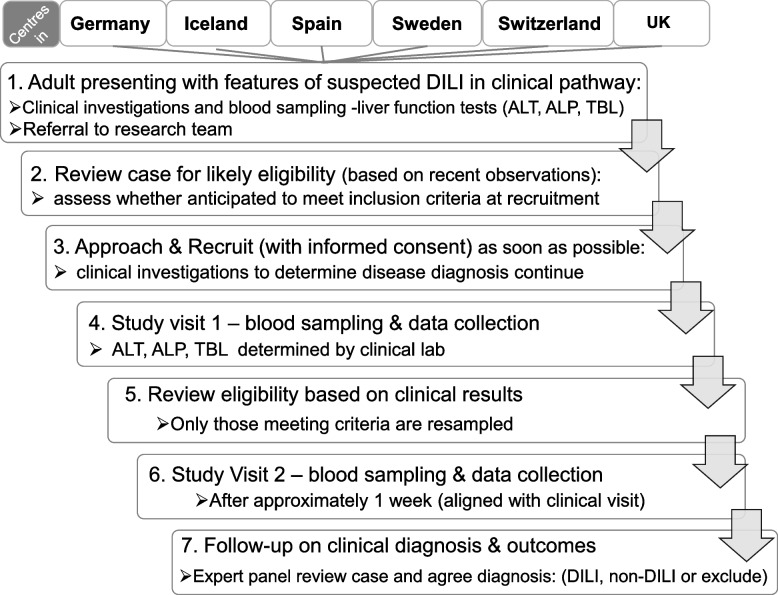
Study procedures. Overview of the TransBioLine study recruitment pathway for suspected drug-induced liver injury (DILI) cases

Patients recruited are those who meet the criteria for DILI, as defined by Aithal et al. [18] and endorsed by the EASL DILI guidelines [8] (Table 2). Patients with acute liver injury where there is a suspicion of DILI and who meet the inclusion criteria are consecutively invited to participate in the study. Suitable patients are identified through referral from clinicians in the recruiting centres. Patients are assessed clinically including investigations. Cases are followed up until recovery, transplantation or death. Once clinical follow-up is completed, the case is centrally reviewed and classified as confirmed DILI or non-DILI acute liver injury arising as a result of alternate causes explaining the clinical manifestation including but not limited to the following: acute viral hepatitis, acute autoimmune hepatitis not previously diagnosed and unrelated to the drug, hypoxic hepatitis or acute biliary obstruction. The study pathway is outlined in Fig. 2.

Blood samples are taken at presentation and follow-up after approximately 7 days, coinciding with clinical care visits. Demographics and lifestyle data are also captured at recruitment (Table 3). The suspected causal agent is considered along with all other current medications and comorbidities. The symptoms and nature of the adverse event are recorded. To enable correct adjudication of the enrolled cases (DILI and non-DILI controls), additional information, such as extended medical history, imaging tests, viral serology tests, autoantibody serology and immunoglobulin levels and histological findings for cases in which biopsy has been performed, is also collected. Each case is followed until resolution or death, when possible. Outcomes are recovery, death (liver related or unrelated) or liver transplantation as determined by the referring clinician. Adjudication (as DILI cases or acute non-DILI controls) as well as outcome is completed prior to biomarker analysis. Where outcomes are not available within the study period, cases of DILI and acute non-DILI controls are excluded from analyses related to the evaluation of prognostic biomarkers.

**Table 3:** Demographic and clinical data collected (where applicable)

Category	Data collected
Demographics	Age at visit, sex at birth, self-reported ethnicity
Anthropometrics	Height, weight, waist circumference
Health status	Diabetes mellitus diagnosis, presence of hypertension, smoking status (cigarettes per week), alcohol intake (gram per week), current pregnancy
Liver episode	Detection date, symptoms, drug/supplement treatments prior to liver episode (dose and duration), whether biopsy was done, outcome
Results of routine clinical investigations: haematology, clinical biochemistry, immunology, and liver function (all with date of testing)	• Total protein, albumin, transferrin, ceruloplasmin, C-reactive protein • Glucose, prothrombin time, international normalised ratio • Urea, creatinine, sodium, potassium • Alanine transaminase (ALT), aspartate transaminase (AST), gamma-glutamyl transferase (GGT), alkaline phosphatase (ALP), total bilirubin (TBL), conjugated bilirubin • Iron, ferritin • Thyroid-stimulating hormone • Lactate dehydrogenase, creatine kinase, creatine kinase-MB • Cholesterol, low-density lipoprotein, high-density lipoprotein, triglycerides • Haemoglobin, platelets, erythrocytes, leukocytes, neutrophils, lymphocytes, monocytes, eosinophils, basophils
Clinical imaging results	Transient elastography, ultrasound
Clinical tests to support diagnosis (routine diagnostics or local practice for clinical care or research)	• Viral serology tests • Autoantibody serology • Immunoglobulin levels • Histological findings for any biopsy performed
Extended clinical information including follow-up information for diagnosis and allocation to study sub-groups	• Comorbidities (past and current) • Concomitant medications, herbal and/or dietary supplements • Past alcohol history • Hospitalisation

Data is captured for drug-induced liver injury cases and nondrug-induced acute liver injury cases and control groups using a standard case report file (CRF) using data from clinical investigations (where available) or from research study visit. Headings are in bold

Details of causality assessment and the adjudication process have been described previously [7]. In brief, investigations are followed by Roussel Uclaf Causality Assessment Method [19] and an expert panel case review including at least 3 experienced clinical hepatologists from 3 different European academic centres. The panel adjudicates episodes as DILI (case) or alternative diseases (acute non-DILI control) or exclude where the investigations are inconclusive or where any disagreement of probable diagnosis occurs within the panel. A sample size of 300 confirmed DILI cases is planned with at least 130 acute non-DILI controls, based on previous studies [15].

### Healthy volunteers

A cohort of 120 adult HV attending 3 visits over 4 weeks are planned to determine the biomarker reference ranges [20]. Exclusion criteria are as follows: BMI > 32 kg/m^2^; aspartate transaminase (AST), alanine transaminase (ALT) level or total bilirubin (TBL) above upper limit of normal (ULN); liver steatosis or fibrosis assessed by FibroScan® transient elastography (controlled attenuation parameter ≥ 260 dB/m after > 4-h fasting) or any known hepatic diseases. Due to the high prevalence of undiagnosed fatty liver disease, it is necessary to ensure that HV have no liver disease as that may influence the levels of candidate biomarkers and could affect the reference interval of the biomarker. For comparison, we have a separate control group with well-defined ARLD and NAFLD to assess this. Participants equally distributed across age ranges 18–40 years, 41–64 years and 65–80 years are planned with an equal male/female (self-reported sex) ratio.

### Chronic liver disease (ARLD/NAFLD)

A cohort of 200 patients diagnosed with chronic liver disease is intended to provide a comparator to assess biomarkers of acute liver injury (DILI in particular) in contrast to common nonviral chronic liver diseases. NAFLD and ARLD are the most common risk factors for raised and fluctuating liver enzymes in the general population [21, 22]. Patients attending secondary care following a clinical diagnosis are recruited. Methods used to confirm the diagnosis of chronic liver disease include liver biopsy (histological evaluation), shear wave elastography (controlled attenuation parameter for evaluation of steatosis and liver stiffness for evaluation of fibrosis) or ultrasound imaging (echo-characteristics of the liver and evidence of portal hypertension). Other chronic liver disease aetiology such as viral hepatitis, metabolic liver diseases (hemochromatosis, alpha-1 antitrypsin deficiency and Wilson’s disease) and autoimmune liver diseases (autoimmune hepatitis, primary biliary cholangitis and primary sclerosing cholangitis) are excluded through combination of standard blood and/or imaging-based tests or liver biopsy as appropriate. Patients with self-reported past/current consumption of alcohol above UK recommended limits (112 g per week for men and women) over past 2 years, or past sustained alcohol excess, are included in the ARLD group and excluded from the NAFLD group (Table 2).

### Psoriasis or rheumatoid arthritis patient cohort

As a further comparator to identify biomarkers that are drug-exposure specific (in the absence of liver injury), we will enroll 100 patients with clinically diagnosed psoriasis or rheumatoid arthritis who are offered methotrexate treatment in clinical practice (Table 2). Methotrexate is an example of a common medication that is associated with the spectrum of acute DILI, adaptation, hyperbilirubinemia and chronic cumulative hepatotoxicity such as liver fibrosis [18, 23]. We will characterise 50 patients on methotrexate therapy who have either liver injury (*n* = 25) or no liver injury (*n* = 25) in addition to 50 patients who have not taken methotrexate but have liver injury (*n* = 25) or without liver injury (*n* = 25). Liver injury will be indicated by liver enzymes (ALT, AST or alkaline phosphatase (ALP)) levels above the ULN or clinically significant fibrosis revealed by biopsy or imaging.

### Data management

A list of demographics and clinical data collected in the case report file (CRF) for the study is outlined in Table 3. For healthy volunteers (HV), only age- and self-reported sex is documented. For chronic liver disease, and psoriasis/rheumatoid arthritis control cohorts demographic, health and clinical information is recorded, along with current medications and comorbidities. The participant details and clinical diagnosis for each case are also reviewed by the lead investigator. The information for recruited participants is stored electronically and held on local secure networks. Case report data is transferred to the Clindex Data Management System (ABX-CRO, Dresden, Germany) where unique study and sample identifiers are created facilitating sharing of pseudo-anonymized data with consortium members through the TranSMART platform (ITTM, Esch-sur-Alzette G.D. Luxembourg) where it is linked with laboratory biomarker analysis results. Study documentation will be monitored by ABX-CRO to ensure ethical compliance and data integrity.

### Sampling and biomarker analysis

**Fig. 3:**
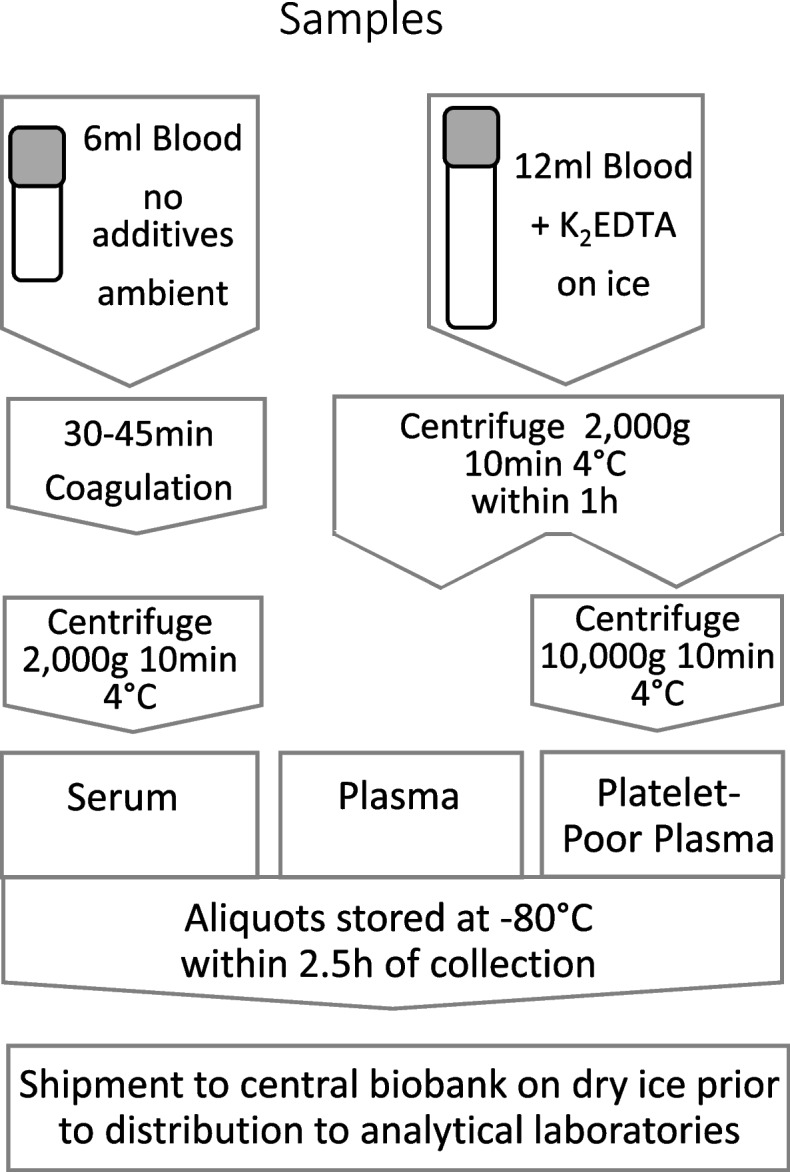
TransBioLine sample processing. Blood is collected at visits in standard clinical blood containers. Link-anonymised aliquots of 0.5 ml are stored in barcoded cryovials at − 80 °C prior to transfer on dry ice to the central biobank (ZeBanC, Charité, Berlin, Germany). ALP, alkaline phosphatase; ALT, alanine transaminase; DILI, drug-induced liver injury; TBL, total bilirubin

Blood samples are collected and processed as outlined in Fig. 3. Samples are stored at − 80 °C in barcode-labelled tubes linked to a unique sample identifier and study participant code. They are shipped to ZeBanC (Charité, Berlin, Germany) and subsequently redistributed to lab facilities. Sample analysers will be blinded to the specific sample group (DILI/non-DILI). An overview of the biomarkers and analytical methods is shown in Table 4.

**Table 4:** Biomarker assays

Biomarker	Sample	Assay method	Laboratory
Alkaline phosphatase (ALP)	Serum		MLM Medical Labs (Mönchengladbach, Germany)
Bilirubin (TBL)	Serum		
Albumin	Serum		
Alanine transaminase (ALT)	Serum		
Glutamate dehydrogenase (GLDH)	Serum		
Cytokeratin-18 full length (K18)	EDTA-plasma	Commercial sandwich immunoassay (Peviva)	SIGNATOPE GmbH (Reutlingen, Germany)
Cytokeratin-18 caspase-cleaved fragment (cc-K18)	EDTA-plasma	Commercial sandwich immunoassay (Peviva)	
*High mobility group box 1 (HMGB1)*	EDTA-plasma	Multiplex immunoprecipitation-nano-liquid chromatography + tandem mass spectrometry (IP-LC–MS/MS) with Orbitrap mass analyzer (Q-Exactive Plus, ThermoFisher)	
***Osteopontin (OPN)***	EDTA-plasma		
Glutamate dehydrogenase (GLDH)	EDTA-plasma		
Macrophage colony-stimulating factor 1 receptor (MCSF1R)	EDTA-plasma		
MicroRNA profile in discovery cohort of 100 DILI and 100 HV	‘Platelet-poor’ EDTA-plasma	Illumina NGS using RealSeq-biofluids serum/plasma miRNA library kit (Somagenics) [24]	TAmiRNA, Vienna, Austria
MicroRNA panel in validation cohort (identified candidate biomarkers from discovery cohort)		Commercial RT-qPCR assay	
Bile acids	EDTA-plasma	Liquid chromatography mass spectrometry (Triple Quad LC/MS, Agilent technologies) [25]	University of Salamanca, Spain
Sphingolipids	EDTA-plasma	Liquid chromatography-mass spectrometry (Q-Exactive HRMS, ThermoFisher) [26]	University of Zurich, Switzerland

Headings are in bold

*Abbreviations*: *LC* Liquid chromatography, *MS* Mass spectrometry, *NGS* Next-generation sequencing, *RT-qPCR* Real-time quantitative polymerase chain reaction. Sample preparation is described in Fig. 3

### Protein analysis

To define the liver injury, established biochemistry tests, ALT, ALP, TBL and albumin, along with glutamate dehydrogenase (GLDH), will be quantified in serum samples using clinical standard assays (Cobas 6000 system, Roche). The candidate protein biomarkers with proposed mechanistic roles in DILI [27] will be quantified from EDTA-plasma without addition of additives or stabilisers (Fig. 3; Table 4). High mobility group box 1 (HMGB1), osteopontin (OPN), macrophage-colony-stimulating factor 1 receptor (MCSF1R) and GLDH will be analysed by multiplex immunoprecipitation coupled to nano-liquid chromatography and tandem mass spectrometry read-out (IP-LC–MS/MS) (Ultimate 300 coupled to Q-Exactive Plus, ThermoFisher) and quantified by adding ^13^C/^15^N -labelled peptide standards to the enzymatically fragmented sample as described by Anselm et al. [28]. The candidate biomarkers cytokeratin-18 (K18) and caspase-cleaved K18 (ccK18) will be determined by sandwich immunoassays as described by the manufacturer (Peviva, Sweden). All the candidate biomarker assays are technically validated according to the FDA evidentiary framework guidelines [29].

### MicroRNA (miRNA) analysis

The miRNA profiles of 100 acute DILI samples and 120 HV will be determined by next-generation sequencing (NGS) of RNA isolated (using either Qiagen miRNeasy Mini kit or Promega Maxwell® RSC miRNA Tissue Kit) from platelet-poor plasma, which has been demonstrated to have necessary stability and assay reproducibility across batches (TAmiRNA, Vienna). A mixture of synthetic, non-mammalian, oligonucleotides (‘miND spike-ins’) will be added to enable the conversion of read counts into absolute values, independent of the library composition and read count, using the established miND bioinformatics pipeline [24]. This will facilitate data comparisons across independent datasets. A cut-off of < 10 read counts will be applied to filter out low-abundant miRNAs with low signal-to-noise ratio. Liver injury-specific markers will be identified through comparison with the healthy volunteer cohort initially, to select ‘signature’ miRNAs having the highest discriminatory power. The signature will be replicated using RT-qPCR to generate data required for computational modelling of multivariate classification algorithms.

### Bile acid analysis

Bile acid profiles in EDTA-plasma from DILI patients and acute non-DILI controls will be identified using liquid chromatography-tandem mass spectrometry (6420 Triple Quad LC/MS, Agilent Technologies, Santa Clara, CA, USA) using an adaptation [25] of a previously described method [30]. Total levels, levels of individual, primary, secondary bile acids and bile acid families will be analysed as well as their ratios (e.g. primary/secondary).

### Sphingolipid analysis

Sphingolipids will be analysed in plasma by LC–MS. For initial untargeted lipidomics, total lipids, extracted in a mixture of methanol:methyl tert-butyl ether:chloroform (4:3:3), will be separated on a C30 LC column using 10-mM ammonium acetate and 0.1% formic acid and either acetonitrile:water (6:4) or isopropanol:acetonitrile (9:1). Eluted lipids will be analysed on a Q Exactive HRMS (ThermoFisher) in a positive and negative mode using a heated electrospray ionisation (HESI). Identification will be based on the predicted mass, isotopic pattern, retention time and specific fragmentation patterns. For quantification of selected sphingoid and deoxysphingoid bases, lipids will be extracted, hydrolyzed, derivatized, separated on a C18 column and analysed as above [26]. Data will be analysed using TraceFinder 4.1 (ThermoFisher).

### Data analysis plan

R (R Core Team) or SAS software (Cary, NC, USA) will be used for statistical analyses. Descriptive statistics, median and interquartile range will be used to describe continuous variables; frequency and percent will be used to describe categorical variables. For consistency, the absolute value of all biomarkers will be log‐transformed. Statistical significance is considered if *p* < 0.05.

### Reference intervals

Reference intervals (RI) will be established to assess the variability of each biomarker measured within (using longitudinal measures for all the visits) and across HV to define properties of variability and reproducibility in healthy subjects. The RI will be calculated according to approved guidelines of the Clinical and Laboratory Standards Institute. For normally distributed parameters (i.e. when the normality test *p*-value is > 0.3 on either the raw or logarithmic scale), *RI* = mean + / − 1.96 SD [31]. The inter- and intra-subject coefficients of variations will be calculated to quantify variability over time periods and between individuals. Differences due to sex and age group will be evaluated. If there is evidence of differences among the groups, then separate reference ranges may be constructed. The impact of excluding potential outliers identified using statistical criteria on the RI value will be determined.

### Biomarkers for DILI diagnosis and prognosis

**Fig. 4:**
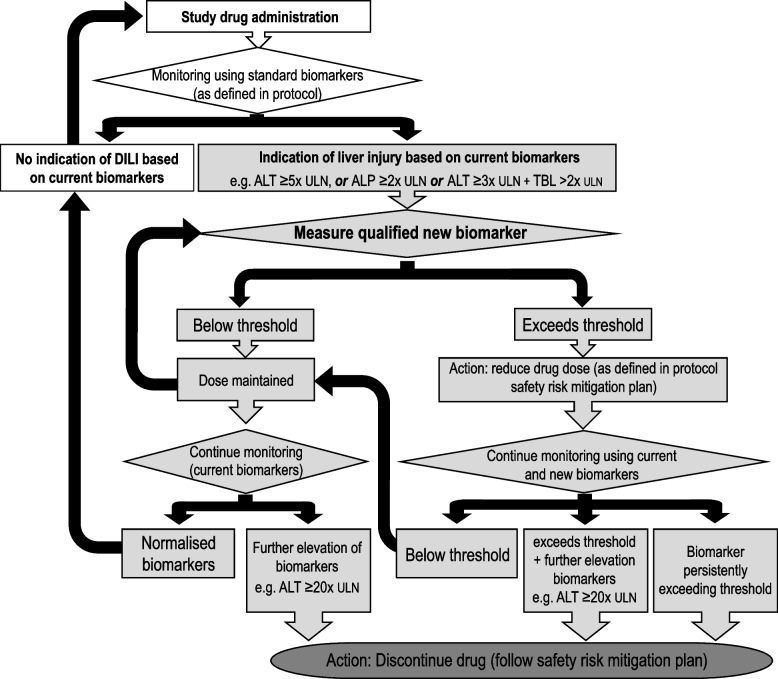
Illustration of possible context of use for a new qualified safety biomarker. The flow diagram shows how adverse effects caused by study drugs in premarketing clinical trials could be monitored using current biomarkers and using new biomarkers (or biomarker panel) to establish whether administrated dose can safely continue or requires reduction. New biomarkers may be incorporated into clinical trials to anticipate early risk for progression of hepatocellular injury to severe DILI in individuals where an initial DILI diagnosis has been established based on elevations of current standard markers (e.g. alanine transaminase (ALT), ≥ 5 × ULN, alkaline phosphatase (ALP) ≥ 2 × ULN or ALT ≥ 3 × ULN and total bilirubin (TBL) > 2 × ULN). If biomarker elevation continues, or increases, the drug would be discontinued according to the safety risk mitigation plan; but if levels normalise, drug administration would continue safely. ULN, upper limit of normal

A context of use will be defined for each biomarker; an example is shown in Fig. 4. Biomarker differences in DILI cases versus non-DILI controls at visit 1, and in the other additional disease control groups (ARLD, NAFLD and systemic diseases), will be determined using one-way ANOVA with Tukey multiple comparison correction. Correlation of each biomarker with ALT will be determined using Pearson’s r coefficients. ROC curve analysis will be utilised to determine each of the candidate biomarkers as well as their combinations for detection of DILI patients and to assess performance of prognostic biomarkers that identify likelihood of a clinical event or disease progression.

The biomarkers will be considered indicative of DILI diagnosis if both the area under the ROC curve (AUC) and the lower end of the 95% confidence interval (CI) are > 0.75 [32]. For the ROC analyses, 100 HV will be used as controls for biomarker qualification. In addition, we will also evaluate the ability of biomarkers (single or multiple in combination) to distinguish DILI from acute non-DILI controls as well as other chronic liver and systemic disease controls. The primary case cohort of interest is the DILI cohort. Multivariate ROC (i.e. using a composite measure of biomarkers) will be based on multiple logistic regression. Hierarchical clustering and principal component analysis (PCA) will also be performed for multivariate analysis and visualisation purposes. The hierarchical clustering technique highlights the similarities of biomarkers expression within and between patients in the same or different groups, while PCA allows us to understand the main variance and to gain insight into high-dimensional biomarkers data.

ROC curve analysis will be used to determine which biomarkers measured at the baseline visit can significantly predict which patients with DILI diagnosis will develop severe DILI/ALF independent of the final outcome (death, liver transplantation or spontaneous recovery) or those who progress to a more severe state during the episode. The ROC analysis will also be applied to traditional biomarkers such as AST and TBL, and their AUCs will be compared with those from candidate biomarkers to see if there is a significant difference in the prognostic value in identifying the development of ALF, transplantation and death.

### Dissemination

The objective of dissemination is to generate visibility and convey the rationale of the project to stakeholders (Table 5). The consortium intends to raise awareness of the project and the findings for improving drug development by increasing efficiency and developing safer medicines.

**Table 5:** Dissemination activities

**Target audiences**	**Reached by**
Consortium members	Electronic communication, newsletter, meetings (virtual and face-2-face)
IMI	Reporting
EFPIA/Industry	Attendance at international conferences or symposia (by networking, poster or oral communications) and using publications
Scientific community (academic and industry)	Attendance at international conferences or symposia (by networking, poster or oral communications) and using publications
Health Policy-makers, Payers, Regulators, Health professionals, Patient Federations	TransBioLine website (www.Transbioline.com), the general media, and articles in specialised journals.
General public including patients	Media and local engagement activities

Headings are in bold

*Abbreviations:* *EFPIA* European Federation of Pharmaceutical Industries and Associations, *IMI* Innovative Medicines Initiative

## Discussion

### Qualification of previously identified biomarkers

This project builds on a previous effort undertaken by the SAFE-T consortium in collaboration with the Predictive Safety Testing Consortium and DILIN Network, to qualify novel DILI protein biomarkers that did not reach full qualification during the lifespan of the SAFE-T consortium [15]. Six of the proteins identified in SAFE-T as potential candidate biomarkers will be analysed further in the current study, to determine their potential to be fully qualified for use as DILI diagnostic and prognostic biomarkers that can provide better identification of liver profile elevations during drug development and in clinical practice. The biomarker potential of these protein candidate biomarkers has also been reported by independent studies. For example, a multivariate model including GLDH and K18 in conjunction with miR-122 was able to detect intrinsic DILI caused by acetaminophen better than individual biomarkers alone [33]. However, limited evaluations and validations are available on the effect of these candidate biomarkers in idiosyncratic DILI and particularly their ability to distinguish DILI from other forms of liver injury, a critical gap that this study intends to fill.

### Identification of new biomarkers

This study will also explore the potential of bile acids, sphingolipids and miRNAs as new DILI biomarkers, individually or combined. Sphingolipids are abundant in hepatocytes, and changes in hepatic levels have been reported in response to drug toxicity [34]. Numerous sphingolipid species can be quantified in plasma, suggesting potential as disease biomarkers. In a model of drug hepatotoxicity, human primary hepatocytes exposed to different acetaminophen concentrations have been reported to differ from control cells in the level of various sphingolipids, such as ceramide and sphingomyelin [35]. Differences in sphingolipid profiles and potential for distinction of *Polygonum multiflorum* DILI from alternate acute liver injury diagnoses have been described in a small cross-sectional cohort [36].

Similarly, the utility of bile acids as biomarkers, both individually and in combinations, has previously been demonstrated in several studies comparing patients with liver injury, liver impairment and HV [37–40]. Serum samples from patients with acetaminophen-induced liver injury have been reported to display a distinct profile of individual bile acids, including increased glycodeoxycholic acid and decreased taurocholic acid levels, compared to other forms of liver injury [40]. Bile acids, metabolised from cholesterol in the liver, are the main constituent of bile, being conjugated in the liver to form primary bile acids or metabolised by gut microbes to form secondary bile acids. Circulating levels become elevated as a consequence of impaired hepatic bile acid uptake, bile acid transport or canalicular functioning. A recent systematic review reported ‘a lack of solid evidence to support the use of individual bile acids or bile acid ratios as biomarkers of liver injury’ [41]. However, their analysis found that bile acids did have a prognostic utility in intrahepatic cholestasis of pregnancy. Prognostic potential of bile acids in acetaminophen DILI was revealed by comparison with cholestatic liver injury patients where severity of liver damage correlated with observed elevation in serum concentrations [39]. Xie et al. have also identified bile acids associated with injury severity and created a predictive model including glycochenodeoxycholic acid, taurochenodeoxycholic acid and norcholic acid associated with severe DILI [38]. Further in-depth studies investigating the potential of bile acids as clinical biomarkers with regard to idiosyncratic DILI are thus merited.

As mediators of many cellular regulatory processes and responses, miRNAs have been investigated and identified as potential organ-specific disease biomarkers. Studies of DILI identified miR-122-5p, highly expressed and predominant in the liver, as a potential hepatotoxicity biomarker [42], and this was evaluated in the SAFE-T cohort [15]. Applications of miRNAs as diagnostic tools to support treatment decisions [43], predict liver function recovery [44] and biomarkers to obtain evidence for intended as well as unintended (adverse or toxic) effects [45, 46] of drug candidates are already underway. An exploratory study on the potential for miRNAs as prognostic DILI biomarkers reported that lower levels of miR-122-5p, -4463 and -4270 are associated with death within 6 months, and that miR-122-5p combined with serum albumin have a sensitivity of 100% for predicting survival [47]. Both miR-122-5p and miR-200a were found to be promising biomarkers for liver injury resulting from antiretroviral therapies [48]. A recent study also reported that combined miR-122 and CK18 biomarkers showed promise in diagnosis of DILI in patients having antituberculosis therapy [49].

### Project strengths and limitations

The potential of this study to identify biomarkers that have clinically-relevant diagnostic and prognostic value would clearly represent a major breakthrough in the management of DILI. This has been recognised by the US FDA, which has accepted this study into its biomarker qualification programme for further qualification of HMGB1, OPN, MCSF1R, GLDH, K18, ccK18, bile acids and sphingolipids, individually or as composite models as part of the TransBioLine Consortium DILI work package, with miRNAs expected to be subsequently added [50].

In this study, our approach is to use biomarkers to ‘detect’ signals that identify acute liver injury followed by investigations to establish the diagnosis of DILI. Church et al. reported AUCs of 0.99, 0.902 and 0.857, for performance in DILI detection for ALT, ALP and TBL, respectively [15]. These biomarkers when used as prognostic markers for death/liver transplantation had AUCs of 0.606, 0.597 and 0.821, respectively, in the same study. These authors did not investigate biomarker performance in differentiating DILI from alternate causes. A recent study found that traditional biomarkers (ALT, ALP, AST and TBL) had AUCs of 0.53–0.65 for distinguishing DILI from ‘non-DILI’ cases [51]. Therefore, currently, diagnosis of DILI is secured through exclusion of alternative aetiology through extensive investigations and a causality assessment process. This approach has been referred to as ‘screen and confirm’, where liver enzymes, as biomarkers with high sensitivity and utility in clinical and trial settings to detect the initial injury signal, are used as a screening modality [52]. New biomarkers are needed to ‘confirm or refute’ the diagnosis of DILI to enable drug administration to continue or stop as appropriate for patient safety (Fig. 4). Therefore, the design of our study is intended to identify biomarkers with application as a second step in the confirmation of diagnosis of DILI among cases of acute liver injury identified using current liver enzyme tests. This nested case–control design with enrolment of cases of acute liver injury and classification of these as DILI cases and acute non-DILI control groups mimics the clinical context of use and can be used for diagnostic as well as prognostic accuracy analyses [15, 51]. DILI cases with information regarding the outcome, recover, death or transplantation are used for the prognostic biomarkers. Since we rely on traditional biomarkers for identification of DILI cases, one disadvantage of this study design is that any biomarkers which are earlier indicators of liver injury than the traditional biomarkers may not be identified and would need to be utilised as parallel tests in the context of use.

The strength of this study is the planned recruitment of a large cohort of patients with extensive phenotyping and follow-up and the rigorous distinction of DILI from possible alternate cause through case adjudication by an expert panel of experienced hepatologists and clinical pharmacologists. The enrolment is integrated within the clinical pathways, and the case–control data therefore closely reflects clinical practice. However, considering the fact that DILI is rare, and the majority of cases recover without serious consequences, there is likely to be only a small number of severe cases, such as those with acute liver failure requiring liver transplantation. This may limit the statistical power of the study, in particular when assessing the prognostic value of the biomarkers in the evaluation of potentially serious outcomes. Since many different drugs, acting via different pathways, can result in DILI, our cohort will have heterogeneity. Furthermore, idiosyncratic DILI likely involves multiple mechanisms, and a potential risk of the current approach is that not all putative mechanisms of DILI are reflected in the biomarkers under study, such that certain forms of DILI may not be picked up by the current biomarkers. This is particularly pertinent for new drugs undergoing clinical trials. However, the provision of long-term biobanked samples provides a legacy available to test future biomarkers.

## Conclusion

The TransBioLine DILI biomarker study is a concerted international effort to enable the exploration and confirmation of new diagnostic and prognostic biomarkers for DILI, by enabling biomarker discovery and validation. If successful, biomarkers developed will help mitigate safety issues during drug development and in clinical practice and will accelerate drug development. Continued collaboration will be necessary to recruit a sufficient number of cases to perform specific drug sub-group analyses and test emerging biomarkers. Establishment of a bioresource and data warehouse facility will support long-term partnerships for biomarker qualification.

## Data Availability

No data is presented. The template case report file used to collect data and sample processing lab manuals are available from the corresponding author on request.

## References

[1] Vega M, Verma M, Beswick D, Bey S, Hossack J, Merriman N (2017). The incidence of drug- and herbal and dietary supplement-induced liver injury: preliminary findings from gastroenterologist-based surveillance in the population of the state of Delaware. Drug Saf.

[2] Bjornsson ES, Bergmann OM, Bjornsson HK, Kvaran RB, Olafsson S (2013). Incidence, presentation, and outcomes in patients with drug-induced liver injury in the general population of Iceland. Gastroenterology.

[3] Shen T, Liu Y, Shang J, Xie Q, Li J, Yan M (2019). Incidence and etiology of drug-induced liver injury in Mainland China. Gastroenterology.

[4] Suzuki A, Tillmann H, Williams J, Hauser RG, Frund J, Suzuki M (2023). Assessment of the frequency, phenotypes, and outcomes of acute liver injury associated with amoxicillin/clavulanate in 1.4 million patients in the Veterans Health Administration. Drug Safety.

[5] Jiang F, Yan H, Liang L, Du J, Jin S, Yang S (2021). Incidence and risk factors of anti-tuberculosis drug induced liver injury (DILI): large cohort study involving 4652 Chinese adult tuberculosis patients. Liver Int.

[6] Reuben A, Tillman H, Fontana RJ, Davern T, McGuire B, Stravitz RT (2016). Outcomes in adults with acute liver failure between 1998 and 2013: an observational cohort study. Ann Intern Med.

[7] Björnsson ES, Stephens C, Atallah E, Robles-Diaz M, Alvarez-Alvarez I, Gerbes A (2023). A new framework for advancing in drug-induced liver injury research. The Prospective European DILI Registry. Liver Int.

[8] EASL Clinical practice guidelines (2019). drug-induced liver injury. J Hepatol.

[9] Onakpoya IJ, Heneghan CJ, Aronson JK (2016). Post-marketing withdrawal of 462 medicinal products because of adverse drug reactions: a systematic review of the world literature. BMC Med.

[10] Stevens JL, Baker TK (2009). The future of drug safety testing: expanding the view and narrowing the focus. Drug Discovery Today.

[11] Kullak-Ublick GA, Andrade RJ, Merz M, End P, Benesic A, Gerbes AL (2017). Drug-induced liver injury: recent advances in diagnosis and risk assessment. Gut.

[12] Koido M, Kawakami E, Fukumura J, Noguchi Y, Ohori M, Nio Y (2020). Polygenic architecture informs potential vulnerability to drug-induced liver injury. Nat Med.

[13] Aithal GP, Rawlins MD, Day CP (1999). Accuracy of hepatic adverse drug reaction reporting in one English health region. BMJ.

[14] M'Kada H, Perazzo H, Munteanu M, Ngo Y, Ramanujam N, Fautrel B (2012). Real time identification of drug-induced liver injury (DILI) through daily screening of ALT results: a prospective pilot cohort study. PLoS ONE.

[15] Church RJ, Kullak-Ublick GA, Aubrecht J, Bonkovsky HL, Chalasani N, Fontana RJ (2019). Candidate biomarkers for the diagnosis and prognosis of drug-induced liver injury: an international collaborative effort. Hepatology.

[16] Biomarker(s) FLoSfD-ILID. [03/02/2022]. Available from: https://www.fda.gov/downloads/Drugs/DevelopmentApprovalProcess/UCM517355.pdf. 2016. Accessed 10 Jan 2021.

[17] Atallah E, Freixo C, Alvarez-Alvarez I, Cubero FJ, Gerbes AL, Kullak-Ublick GA (2021). Biomarkers of idiosyncratic drug-induced liver injury (DILI) - a systematic review. Expert Opin Drug Metab Toxicol.

[18] Aithal GP, Watkins PB, Andrade RJ, Larrey D, Molokhia M, Takikawa H (2011). Case definition and phenotype standardization in drug-induced liver injury. Clin Pharmacol Ther.

[19] Danan G, Benichou C (1993). Causality assessment of adverse reactions to drugs–I. A novel method based on the conclusions of international consensus meetings: application to drug-induced liver injuries. J Clin Epidemiol.

[20] https://www.fda.gov/patients/drug-development-process/step-3-clinical-research. Accessed 10 Jan 2021.

[21] Sherwood P, Lyburn I, Brown S, Ryder S (2001). How are abnormal results for liver function tests dealt with in primary care? Audit of yield and impact. BMJ.

[22] Armstrong MJ, Houlihan DD, Bentham L, Shaw JC, Cramb R, Olliff S (2012). Presence and severity of non-alcoholic fatty liver disease in a large prospective primary care cohort. J Hepatol.

[23] Aithal GP (2011). Hepatotoxicity related to antirheumatic drugs. Nat Rev Rheumatol.

[24] Khamina K, Diendorfer AB, Skalicky S, Weigl M, Pultar M, Krammer TL (2022). A MicroRNA Next-Generation-Sequencing Discovery Assay (miND) for Genome-Scale Analysis and Absolute Quantitation of Circulating MicroRNA Biomarkers. Int J Mol Sci.

[25] Nytofte NS, Serrano MA, Monte MJ, Gonzalez-Sanchez E, Tumer Z, Ladefoged K (2011). A homozygous nonsense mutation (c.214C->A) in the biliverdin reductase alpha gene (BLVRA) results in accumulation of biliverdin during episodes of cholestasis. J Med Genet.

[26] Penno A, Reilly MM, Houlden H, Laurá M, Rentsch K, Niederkofler V (2010). Hereditary sensory neuropathy type 1 is caused by the accumulation of two neurotoxic sphingolipids. J Biol Chem.

[27] Andrade RJ, Chalasani N, Björnsson ES, Suzuki A, Kullak-Ublick GA, Watkins PB (2019). Drug-induced liver injury. Nat Rev Dis Primers.

[28] Anselm V, Sommersdorf C, Carrasco-Triguero M, Katavolos P, Planatscher H, Steinhilber A (2021). Matrix and sampling effects on quantification of protein biomarkers of drug-induced liver injury. J Proteome Res.

[29] https://www.fda.gov/media/119271/download. Accessed 10th Jan 2021.

[30] Ye L, Liu S, Wang M, Shao Y, Ding M (2007). High-performance liquid chromatography-tandem mass spectrometry for the analysis of bile acid profiles in serum of women with intrahepatic cholestasis of pregnancy. J Chromatogr, B: Anal Technol Biomed Life Sci.

[31] Horowitz GL, Altaie S, Boyd JC, Ceriotti F, Garg U, Horn P (2008). Defining, establishing, and verifying reference intervals in the clinical laboratory: approved guideline, 3rd ed ed. Clinical, Laboratory Standards Institue.

[32] Hosmer DW, Lemeshow S. Applied logistic regression. New York: Wiley; 2000.

[33] Llewellyn HP, Vaidya VS, Wang Z, Peng Q, Hyde C, Potter D (2021). Evaluating the sensitivity and specificity of promising circulating biomarkers to diagnose liver injury in humans. Toxicol Sci.

[34] Qu L, Qu F, Jia Z, Wang C, Wu C, Zhang J (2015). Integrated targeted sphingolipidomics and transcriptomics reveal abnormal sphingolipid metabolism as a novel mechanism of the hepatotoxicity and nephrotoxicity of triptolide. J Ethnopharmacol.

[35] Li L, Wang H, Jones JW (2020). Sphingolipid metabolism as a marker of hepatotoxicity in drug-induced liver injury. Prostaglandins Other Lipid Mediat.

[36] Huang Y, Zhao X, Zhang ZT, Chen SS, Li SS, Shi Z (2020). Metabolomics profiling and diagnosis biomarkers searching for drug-induced liver injury implicated to Polygonum multiflorum: a cross-sectional cohort study. Front Med.

[37] Ma Z, Wang X, Yin P, Wu R, Zhou L, Xu G (2019). Serum metabolome and targeted bile acid profiling reveals potential novel biomarkers for drug-induced liver injury. Medicine (Baltimore).

[38] Xie Z, Zhang L, Chen E, Lu J, Xiao L, Liu Q (2021). Targeted metabolomics analysis of bile acids in patients with idiosyncratic drug-induced liver injury. Metabolites.

[39] Woolbright BL, McGill MR, Staggs VS, Winefield RD, Gholami P, Olyaee M (2014). Glycodeoxycholic acid levels as prognostic biomarker in acetaminophen-induced acute liver failure patients. Toxicol Sci.

[40] Luo L, Aubrecht J, Li D, Warner RL, Johnson KJ, Kenny J (2018). Assessment of serum bile acid profiles as biomarkers of liver injury and liver disease in humans. PLoS ONE.

[41] Azer SA, Hasanato R (2021). Use of bile acids as potential markers of liver dysfunction in humans: a systematic review. Medicine.

[42] Liu Y, Li P, Liu L, Zhang Y (2018). The diagnostic role of miR-122 in drug-induced liver injury: a systematic review and meta-analysis. Medicine (Baltimore).

[43] Hackl M, Heilmeier U, Weilner S, Grillari J (2016). Circulating microRNAs as novel biomarkers for bone diseases - complex signatures for multifactorial diseases?. Molecular and CellularEndocrinology.

[44] Starlinger P, Hackl H, Pereyra D, Skalicky S, Geiger E, Finsterbusch M (2019). Predicting postoperative liver dysfunction based on blood-derived MicroRNA signatures. Hepatology.

[45] Krauskopf J, de Kok TM, Schomaker SJ, Gosink M, Burt DA, Chandler P (2017). Serum microRNA signatures as “liquid biopsies” for interrogating hepatotoxic mechanisms and liver pathogenesis in human. PLoS ONE.

[46] Schraml E, Hackl M, Grillari J (2017). MicroRNAs and toxicology: a love marriage. Toxicol Rep.

[47] Russo MW, Steuerwald N, Norton HJ, Anderson WE, Foureau D, Chalasani N (2017). Profiles of miRNAs in serum in severe acute drug induced liver injury and their prognostic significance. Liver Int.

[48] Murray DD, Suzuki K, Law M, Trebicka J, Neuhaus Nordwall J, Johnson M (2017). Circulating miR-122 and miR-200a as biomarkers for fatal liver disease in ART-treated, HIV-1-infected individuals. Sci Rep.

[49] Rupprechter SAE, Sloan DJ, Oosthuyzen W, Bachmann TT, Hill AT, Dhaliwal K (2021). MicroRNA-122 and cytokeratin-18 have potential as a biomarkers of drug-induced liver injury in European and African patients on treatment for mycobacterial infection. Br J Clin Pharmacol.

[50] https://www.fda.gov/media/151078/download. Accessed 21 Jan 2021.

[51] Ravindra KC, Vaidya VS, Wang Z, Federspiel JD, Virgen-Slane R, Everley RA (2023). Tandem mass tag-based quantitative proteomic profiling identifies candidate serum biomarkers of drug-induced liver injury in humans. Nat Commun.

[52] Vazquez JH, McGill MR (2021). Redrawing the map to novel DILI biomarkers in circulation: where are we, where should we go, and how can we get there?. Livers.

